# High‐density lipoproteins alleviate the endotoxin burden in patients with peritonitis and sepsis: The LIPS study

**DOI:** 10.1111/eci.70099

**Published:** 2025-07-19

**Authors:** Maxime Nguyen, Marvin Alvarez, Vivien Berthoud, Gaetan Pallot, Sohel Abagri, Damien Leleu, Jean‐Paul Pais‐De‐Barros, Pablo Ortega‐Deballon, Pierre‐Grégoire Guinot, David Masson, Thomas Gautier, Belaid Bouhemad

**Affiliations:** ^1^ Department of Anesthesiology and Intensive Care Dijon University Hospital Dijon France; ^2^ Université Bourgogne Europe Dijon France; ^3^ Center for Translational and Molecular Medicine (CTM), INSERM UMR1231, Lipness Team Dijon France; ^4^ Laboratory of Clinical Chemistry Dijon University Hospital Dijon France; ^5^ Lipidomic Facility, Université de Bourgogne Dijon France; ^6^ Department of Digestive and Oncological Surgery Dijon‐Bourgogne University Hospital Dijon France

**Keywords:** critical care, endotoxemia, inflammation, lipoproteins, sepsis, septic‐shock

## Abstract

**Background:**

The high‐density lipoprotein (HDL) and the phospholipid transfer protein (PLTP) have been demonstrated to enhance endotoxin elimination and inactivation in animal models of sepsis. This study aimed to confirm such a role in patients presenting with abdominal sepsis undergoing emergent surgery and explore the relationships between HDL, PLTP and the lipopolysaccharide (LPS) burden (mass and activity).

**Methods:**

Patients operated for abdominal sepsis were prospectively included in the study. Blood samples were obtained before surgery, at the end of the operation (H0), 4 h (H4) and 24 h (H24) later. Peritoneal fluid was also sampled. HDL cholesterol, LDL cholesterol, PLTP activity, LPS mass and activity were measured.

**Results:**

Twenty‐seven patients were included. At H0, LPS mass was mostly measured in the lipoprotein fractions (46% [23; 58] in HDL and 36% [29; 54] in LDL). Overall, LPS mass and LPS activity did not decrease in the 24 h following admission to the ICU. Both HDLc concentrations and PLTP activity were associated with increased H4‐LPS elimination (drop in LPS concentration, −3% [−26;10] vs. 29% [13;52], *p* < 0.01 and −2% [−15;10] vs. 20% [8:52], *p* = 0.03, respectively). Increased H4‐LPS elimination was associated with reduced inflammation (plasma cytokine concentration) and mortality. High HDL cholesterol was associated with reduced mortality but not with inflammation.

**Conclusion:**

Our data support the role of HDL and PLTP in the elimination of LPS during human peritonitis with sepsis. Increased H4‐LPS elimination was associated with reduced inflammation and lower mortality.

ClinicalTrials.gov: NCT04126577.

## Graphical abstract

Our data support the role for HDL and PLTP in the elimnation of LPS during human peritonitis with sepsis. Increased H4‐LPS elimination was associated with reduced inflammation and lower mortality.

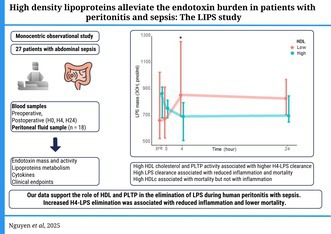

## 1 INTRODUCTION

In patients with secondary peritonitis, the presence of gram‐negative bacteria‐derived lipopolysaccharides (LPS) in the peritoneal cavity is a strong inducer of the host immune response that aims to eliminate pathogens. However, overwhelming inflammation is responsible for organ injury and sepsis.^1^ The failure of randomized controlled trials aiming to blunt LPS immune response^2^ underlines the need for precision medicine, and thus to further understand the endotoxin burden to stratify individual patient risk.

High‐density lipoproteins (HDL) have been studied for their ability to modulate inflammation in animals and patients with sepsis.^3^, ^4^ In particular, low HDL levels are associated with adverse outcomes^5^, ^6^ and recombinant HDL has shown promising preliminary results as a therapeutic for human sepsis.^7^ An important mechanism beyond HDL's protective effect against sepsis is its role in reducing LPS burden. In previous studies, we showed that LPS binding to HDL under the action of phospholipid transfer protein (PLTP) promotes LPS inactivation and elimination.^3^, ^4^, ^8^, ^9^, ^10^, ^11^ This HDL‐driven elimination might start early in the pathological process, within the peritoneal cavity.^11^

Nevertheless, whereas those phenomena have been described under experimental conditions in animal models,^3^, ^10^, ^11^, ^12^ the relationship between lipoproteins and LPS inactivation/elimination during human sepsis is poorly described. Hence, we aimed to investigate the relevance of lipoprotein‐mediated LPS elimination in patients with acute peritonitis and sepsis by analysing the relationships between HDL, PLTP and LPS burden (mass and activity). We hypothesised that, as in experimental studies, HDL and PLTP would promote LPS binding, inactivation and elimination.

## 2 METHODS

### 2.1 Population

A prospective cohort was created in the surgical intensive care unit (ICU) of the University Hospital of Dijon. This research was approved by the institutional review board (CPP Sud‐Est VI, France; ref. 2019‐A02000‐57). Informed consent was obtained from all patients or next of kin before inclusion. This was a convenience cohort; 27 patients were prospectively included between September 2019 and September 2022. Inclusion criteria were as follows: age ≥ 18 years, admission to the operating room for secondary peritonitis (diagnosis suspected by the medico‐surgical team and confirmed by operative surgical findings) with sepsis (qSOFA ≥2 or need for vasopressor or mechanical ventilation). Exclusion criteria were as follows: patients not affiliated to national health insurance, under legal guardianship, pregnant or breastfeeding, with immunosuppression or end‐of‐life decision. The present report was drafted in agreement with the STROBE statement.^13^

### 2.2 Protocol

Demographic data were collected upon ICU admission, and severity scores (sequential organ failure assessment, SOFA, simplified acute physiology score, SAPS 2)^14^, ^15^ were calculated. Clinical data and mortality were collected in the OR and during the ICU stay.

Blood was sampled at 4 time points: preoperative (pre, within the hours before surgery), after surgery (at ICU admission, H0), 4 h after surgery (H4) and 24 h after surgery (H24). Peritoneal fluid was collected during surgery by the surgeon under visual control immediately after opening the peritoneal cavity.

### 2.3 Biological measurements

Blood was collected in EDTA tubes and centrifuged (3000 g, 4 min, 4°C). Plasma was then stored at −80°C before analysis. Peritoneal fluid was collected in dry tubes and was centrifuged (3000 g, 4 min, 4°C). The supernatant was collected and stored at −80°C before analysis.

At the end of the study, those samples were used for biological assays. Each measurement was performed as a single measurement. LPS mass concentration was determined by using liquid chromatography coupled with mass spectrometry (quantitation of esterified 3 hydroxy‐fatty acids with chain length ranging from 10 and 18 carbons), as previously described.^16^ Results were expressed as pmol of 3 hydroxy‐fatty acids (3OH) per ml sample. LPS activity (EU/ml) was measured by LAL (Limulus amebocyte lysate) assay (HycultBiotech Inc., Wayne, USA) according to the manufacturer's instructions. The inactivation of LPS molecules was reflected by dividing the activity value obtained by the LAL assay by LPS mass concentration obtained by mass spectrometry (activity to mass ratios expressed as EU/mmol). Phospholipid transfer protein activity (PLTPa) was measured by fluorescence using a commercially available kit (Merck, Darmstadt, Germany) on a VarioskanLUX microplate reader (ThermoFisher) as previously described.^10^ High‐density lipoprotein cholesterol (HDLc) and low‐density lipoprotein cholesterol (LDLc) concentrations were measured on an Atellica system with dedicated kits (Siemens‐France). Cytokines (IL‐6, IL‐8, IL‐10 and TNF‐α) were measured using a Luminex® Human Magnetic assay (Merck, Darmstadt, Germany) following manufacturer instructions.

The different lipoprotein fractions were isolated from plasma or peritoneal fluid (sample volume, 100 μL) according to their density by sequential ultracentrifugation as previously described.^17^, ^18^ Densities were adjusted by adding controlled volumes of sodium/potassium bromide solutions. Three ultracentrifugation steps were conducted at 100,000 rpm at 4°C in a TLA100.1 rotor with an Optima MAX XP ultracentrifuge (Beckman, Palo Alto, CA) at the appropriate densities to obtain 4 fractions: first step (2‐h run), triglyceride‐rich lipoproteins (*d* < 1.019); second step (3‐h run), LDL (1.019 < *d* < 1.063); third step (5.5‐h run), HDL (1.063 < *d* < 1.21) and lipoprotein‐free infranatant (*d* > 1.21). Sodium and potassium bromide salts were removed from the resulting fractions by dialysis against phosphate‐buffered saline (PBS) solution using 12‐14kD MWCO dialysis membranes. The lowest or highest value of detection was used for parameters with values out of the limit of detection.

### 2.4 Outcomes

The primary outcome was LPS mass. Secondary outcomes were inflammation (measured by cytokines, IL‐6, IL10, TNF‐α and IL‐8) and ICU mortality.

### 2.5 Definitions

The reference time point for LPS measurement was the immediate post‐operative time point (H0), because we considered that the peritoneal cavity was cleared of LPS after surgery, thus limiting further LPS translocation.

LPS concentration or mass refers to LPS quantified by mass spectrometry.

LPS activity refers to LPS biological activity measured by LAL.

The capacity of plasma to inactivate LPS was estimated by calculating the ratio of active LPS (measured by LAL) to LPS mass (measured by mass spectrometry). A low ratio indicates a good capacity to inactivate LPS. The ratio was multiplied by 10^6^ to improve clarity.

LPS variation was calculated as (LPS mass at H4−LPS mass at H0)/LPS mass at H0 and as (LPS mass at H24−LPS mass at H0)/LPS mass at H0.

The binding of LPS to lipoproteins was calculated as (LPS mass in the lipoprotein fraction/LPS mass in total plasma).

### 2.6 Statistical analysis

Due to the exploratory nature of this study, there was no sample size calculation. The number of 40 patients was initially considered as an acceptable compromise between power and feasibility. However, at the end of the inclusion period, 27 patients were recruited in this cohort. Quantitative data were presented as medians and interquartile ranges, and qualitative data as frequencies and percentages. Patients were dichotomised into two groups by the median for HDL cholesterol and PLTP activity measured at ICU admission, and the two groups were compared. In addition, patients alive at ICU discharge were compared to those who did not survive. Due to the low number of subjects, only non‐parametric tests were used. Quantitative data were compared with a non‐parametric test (a Wilcoxon test or a Kruskall–Wallis test), qualitative data were compared with a Fisher exact test and correlations were computed with the Spearman method. Paired data were compared using a paired Wilcoxon test. Statistical significance was defined as a *p*‐value of less than 0.05. *p*‐value were not corrected for multiple testing. Statistical analysis was performed with R. Missing data were omitted.

## 3 RESULTS

### 3.1 Baseline characteristics

Twenty‐seven patients were included in the analysis. Twenty‐seven plasma samples were collected at the pre and H0 time points. Twenty‐six samples were available at H4 and 22 at H24. Eighteen peritoneal fluid samples were analysed. Baseline characteristics are presented in Table 1. Median age was 70.0 [61.5;73.5], 44% of patients were women. At inclusion, the median SOFA score was 8 [5;11] and the median SAPS II score was 55 [48;59]. ICU mortality was 33.3%. Lipid parameters across the different time points are presented in Table S1. The overall LPS mass decreased during surgery (between pre and H0 time points) and was stable during the 24 h following ICU admission. At H0, LPS was mostly located in the lipoprotein fractions (46% [23; 58] in HDL and 36% [29;54] in LDL). Patients with high LPS concentrations in HDL had low LPS concentrations in LDL (*r* = −0.56, *p* < 0.01).

**TABLE 1:** Baseline characteristics.

	All
	*n* = 27
Age (year)	70.0 [61.5;73.5]
Sexe (women)	12 (44.4%)
BMI (kg/m^2^)	27.5 [20.8;28.5]
Medical history	
Myocardial infraction	7 (25.9%)
Stroke	4 (14.8%)
Neoplasia	12 (44.4%)
Hemopathy	1 (3.70%)
Chronic respiratory failure	6 (22.2%)
Chronic renal failure	4 (14.8%)
Cirrhosis	1 (3.70%)
Statins	15 (57.7%)
Indentification (culture)	
Gram‐negative bacillus	9 (32.3%)
Gram‐positive cocci	2 (7.4%)
Both	3 (11.1%)
No identification	13 (48.1%)
Bacteremia	8 (34.7%)
Severity (at inclusion)	
SOFA	8.00 [5.00; 11.0]
SAPS 2	55.0 [47.5; 59.0]
Norepinephrine (ug/kg/min)	0.11 [0; 0.59]
Lactatemia (mmol/L)	2.40 [1.45; 5.38]
Outcome	
ICU LOS	5.00 [4.00; 16.0]
ICU mortality	9 (33.3%)

*Note*: Results are presented as median [Q1;Q3] or number and percentage.

Abbreviations: BMI, body mass index; HDL, high density lipoprotein; ICU, intensive care unit; LDL, low density lipoprotein; LOS, length of stay; SAPS, simplified acute physiology score; SOFA, sequential organ failure assessment.

### 3.2 Increased H4‐LPS elimination and high HDLc were associated with ICU survival

**FIGURE 1:**
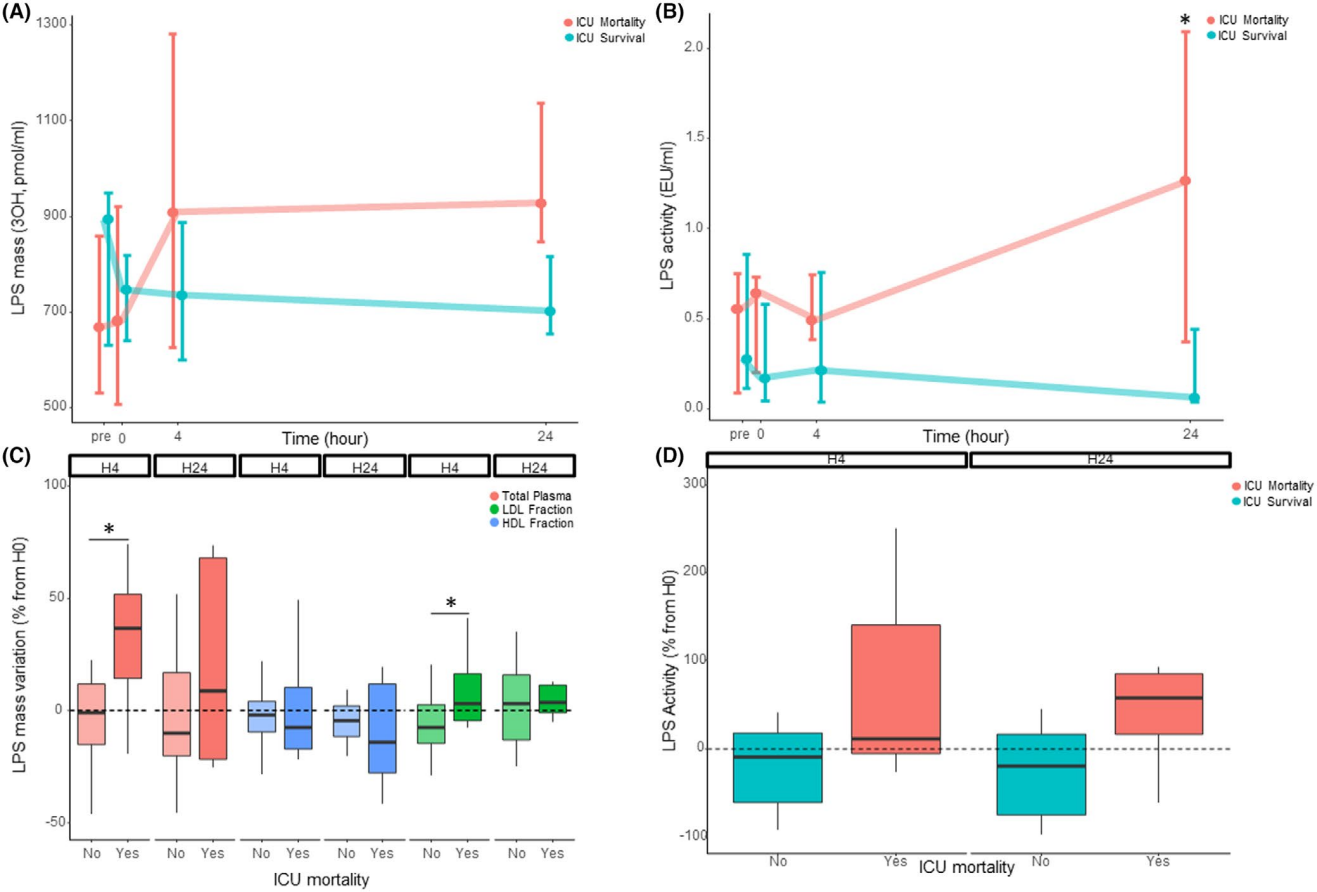
LPS burden depending on intensive care unit mortality. (A) LPS mass in total plasma and in the LDL and HDL fractions. (B) LPS activity (C) LPS mass variations in total plasma and lipoprotein fractions. (D) LPS activity variations in total plasma. HDL, high‐density lipoproteins; LDL, low‐density lipoproteins; LPS, Lipopolysaccharides. Data are presented as median and IQR [25;75]. *Refers to significant differences from H0 (*p* < 0.05, Wilcoxon paired test).

A comparison of plasma LPS mass and activity between ICU survivors and deceased patients is shown in Figure 1. LPS mass tended to be higher (Figure 1A) and LPS activity was significantly increased at 24 h in deceased patients when compared with survivors (Figure 1B). When comparing the variations of LPS levels with those reported at H0, ICU survival was associated with increased 4‐h plasma LPS elimination (−1% [−15;12] vs. 37% [14:52], *p* = 0.023) (Figure 1C). In addition, ICU survivors had lower initial cytokine concentrations (Figure S1) and higher HDL cholesterol at ICU admission (0.64 mmol/L [0.36;0.85] vs 0.30 mmol/L [0.23;0.35] *p* = 0.035) (Figure S2). Bacteremia and bacterial identifications were not associated with LPS mass or activity (Tables S2 and S3).

### 3.3 Patients with high HDLc and patients with high PLTP activity had increased H4‐LPS elimination

**FIGURE 2:**
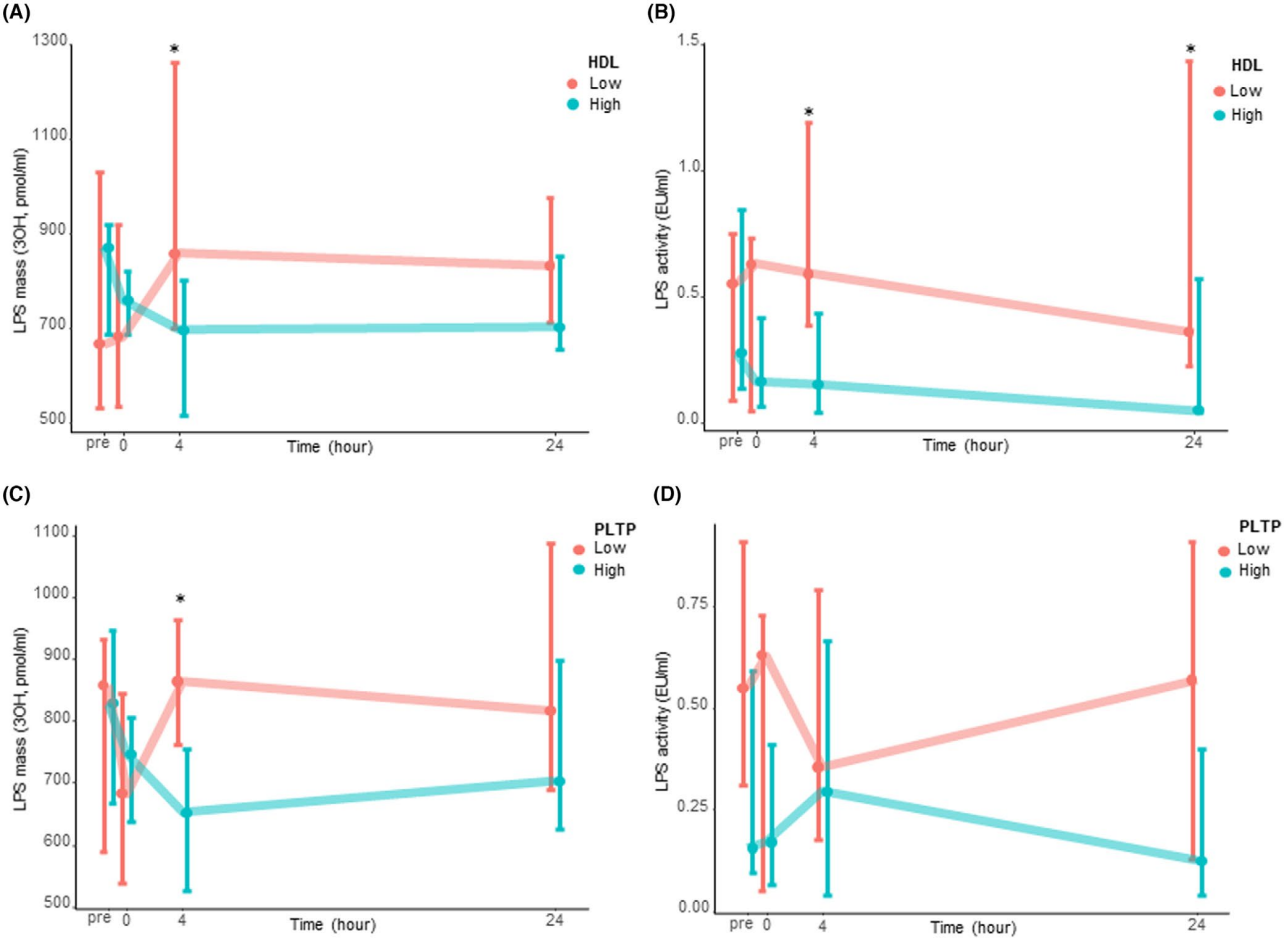
LPS burden depending on HDL cholesterol concentration and PLTP activity. Groups were dichotomized by the median HDLc (panels A, B) or PLTP activity (panels C, D). (A, C) LPS mass in total plasma. (B, D) LPS activity in total plasma. HDL, high‐density lipoproteins; LPS, lipopolysaccharides; PLTP, phospholipid transfer protein. Data are presented as median and IQR [25; 75]. *Refers to significant between groups differences (*p* < 0.05, Wilcoxon tests, no correction for repeated testing).

Patients were dichotomized according to the median HDLc and PLTP value at H0. Four hours after ICU admission, LPS concentrations were lower in patients with high HDLc and PLTP activity (Figure 2A,C). LPS activity was lower in patients with high HDLc at 4 and 24 h (Figure 2B). There was no difference in LPS activity in patients with high PLTP activity (Figure 2D).

**FIGURE 3:**
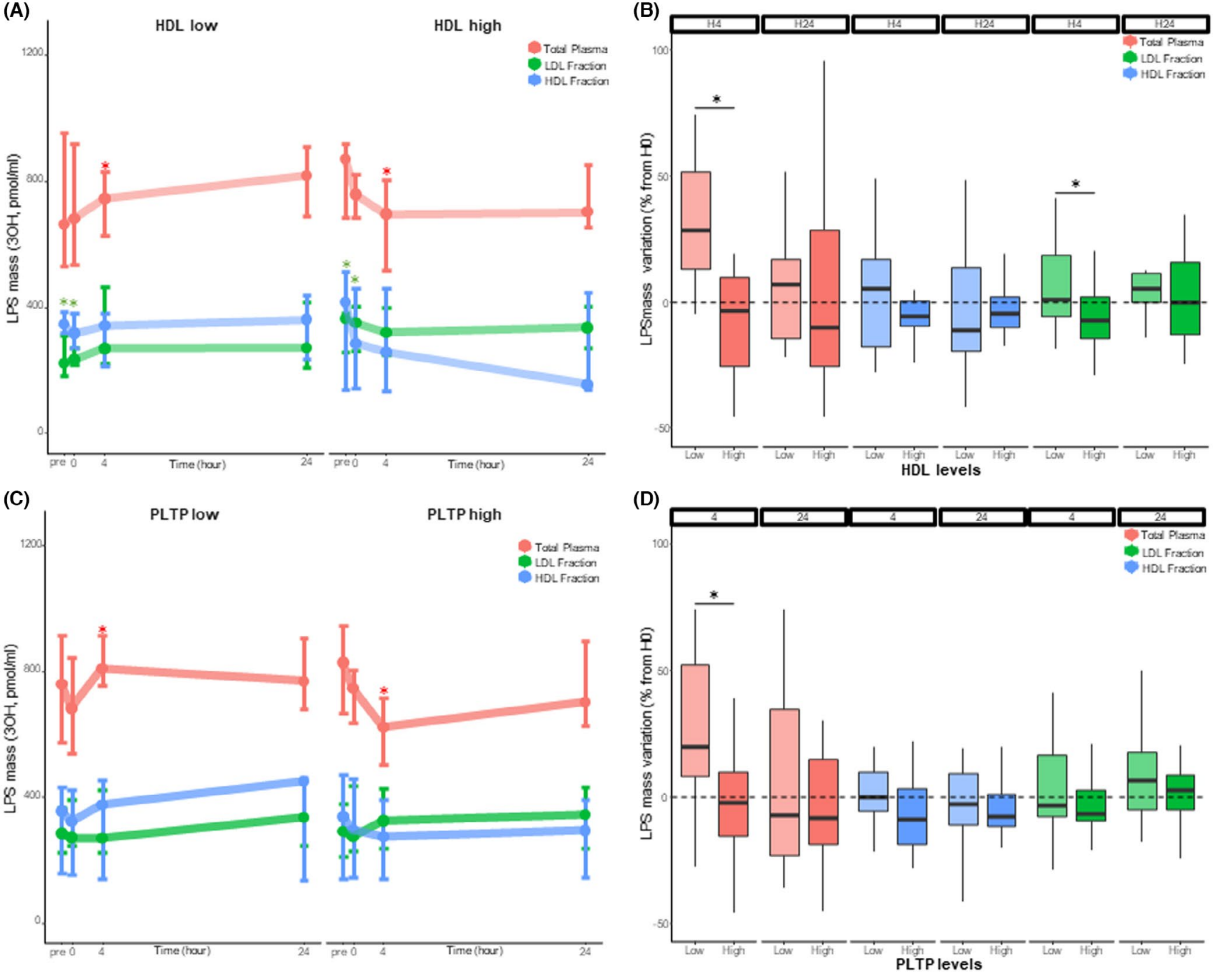
LPS concentration, distribution and elimination in total plasma, HDL and LDL fractions depending on the level of HDLc (panels A, B) and PLTP (panels C, D). (A, C) LPS mass in total plasma, HDL and LDL fractions. (B, D) LPS elimination in total plasma, in the HDL and LDL fractions. Groups were dichotomized by the median HDLc or PLTP activity value. Concentrations were compared between high and low conditions. There were no significant differences. HDL, high‐density lipoproteins; LPS, lipopolysaccharides; PLTP, phospholipid transfer protein. Data are presented as median and IQR [25; 75]. *Refers to significant between groups differences (*p* < 0.05, Wilcoxon tests, no correction for repeated testing).

Patients with high HDLc and patients with high PLTP activity had higher 4‐h LPS elimination (−3% [−26;10] vs. 29% [13;52], *p* < 0.01 and −2% [−15;10] vs. 20% [8:52], *p* = 0.02 respectively, Figure 3B,D). In patients with high HDLc, LPS elimination was higher in the LDL fraction (Figure 3B). Patients with high HDLc had a higher decrease in LPS activity between H0 and H4 (Figure S3). At H24, neither high HDLc nor high PLTP activity was associated with LPS concentration or elimination.

High HDLc was associated with a higher LPS mass in LDL before surgery and at H0. PLTP activity was not associated with higher binding of LPS to HDL (Figure 3A,C).

### 3.4 LPS measured in the HDL fraction of plasma and peritoneal fluid were correlated

**FIGURE 4:**
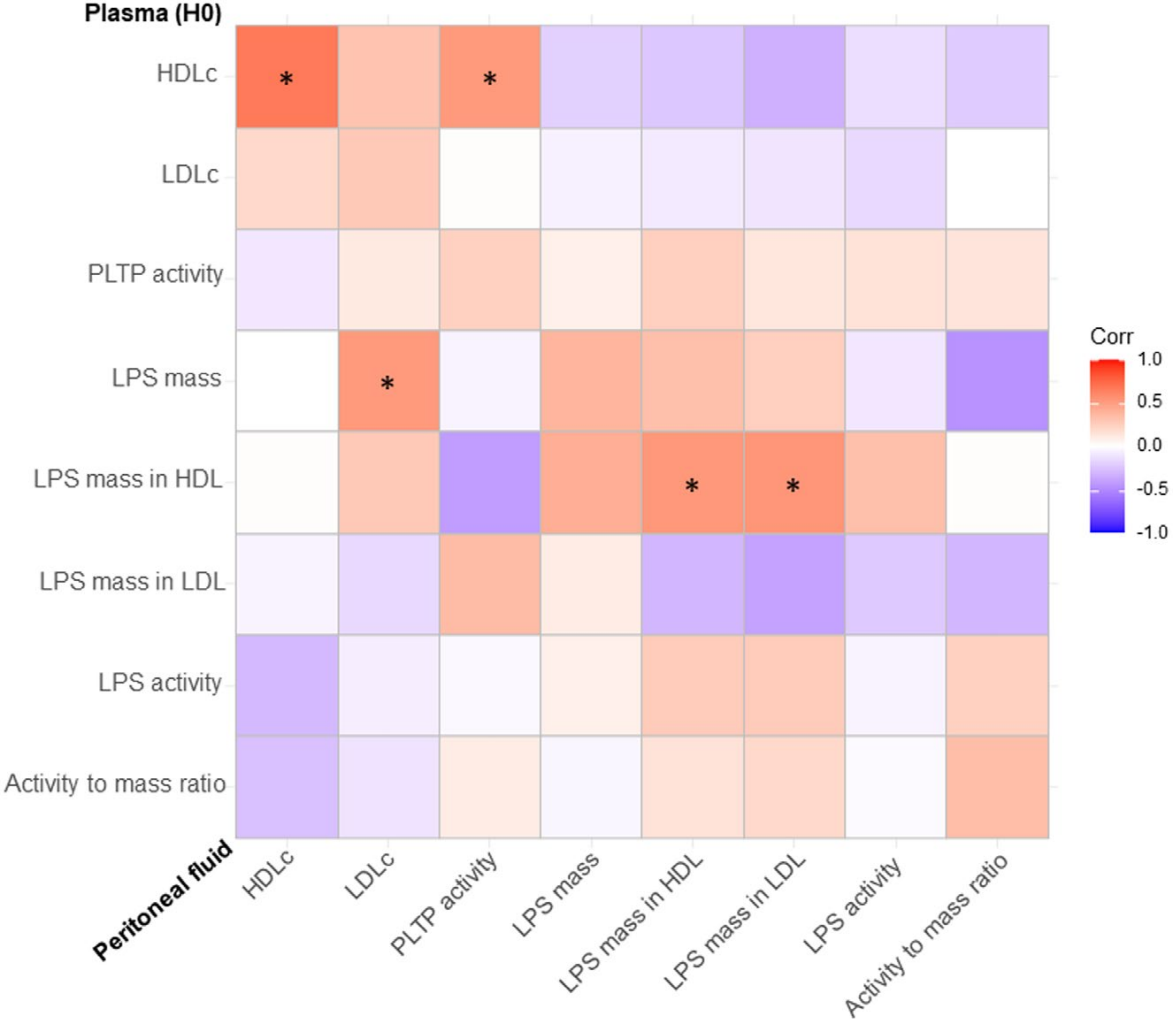
Correlations between plasma and peritoneal fluid regarding lipoproteins, PLTP activity and the LPS burden. HDL, high density lipoproteins; LDL, low density lipoproteins; PLTP, phospholipid transfer protein; LPS, lipopolysaccharides. *Refers to significant correlations (*p* < 0.05, spearman method, no correction for multiple testing).

Ten and 4 patients out of 18 patients had respectively HDL and LDL cholesterol below the lowest limit of detection in peritoneal fluid. Correlations between plasma and peritoneal fluid content in lipoproteins and LPS are presented in Figure 4. High LPS concentration in the HDL fraction in plasma correlates with high LPS concentration in the HDL and LDL fraction in peritoneal fluid (0.53 *p* = 0.03 and 0.54 *p* = 0.02, respectively). High plasma HDLc was associated with high peritoneal HDLc (*r* = 0.67, *p* < 0.01) and with increased intraperitoneal PLTP activity (*r* = 0.52, *p* = 0.03). However, plasma HDLc and PLTP activity were not associated with lower LPS mass or activity, or with a higher capacity to inactivate LPS in the peritoneal fluid (Figure 4).

### 3.5 Increased H4‐LPS elimination was associated with reduced inflammation

**FIGURE 5:**
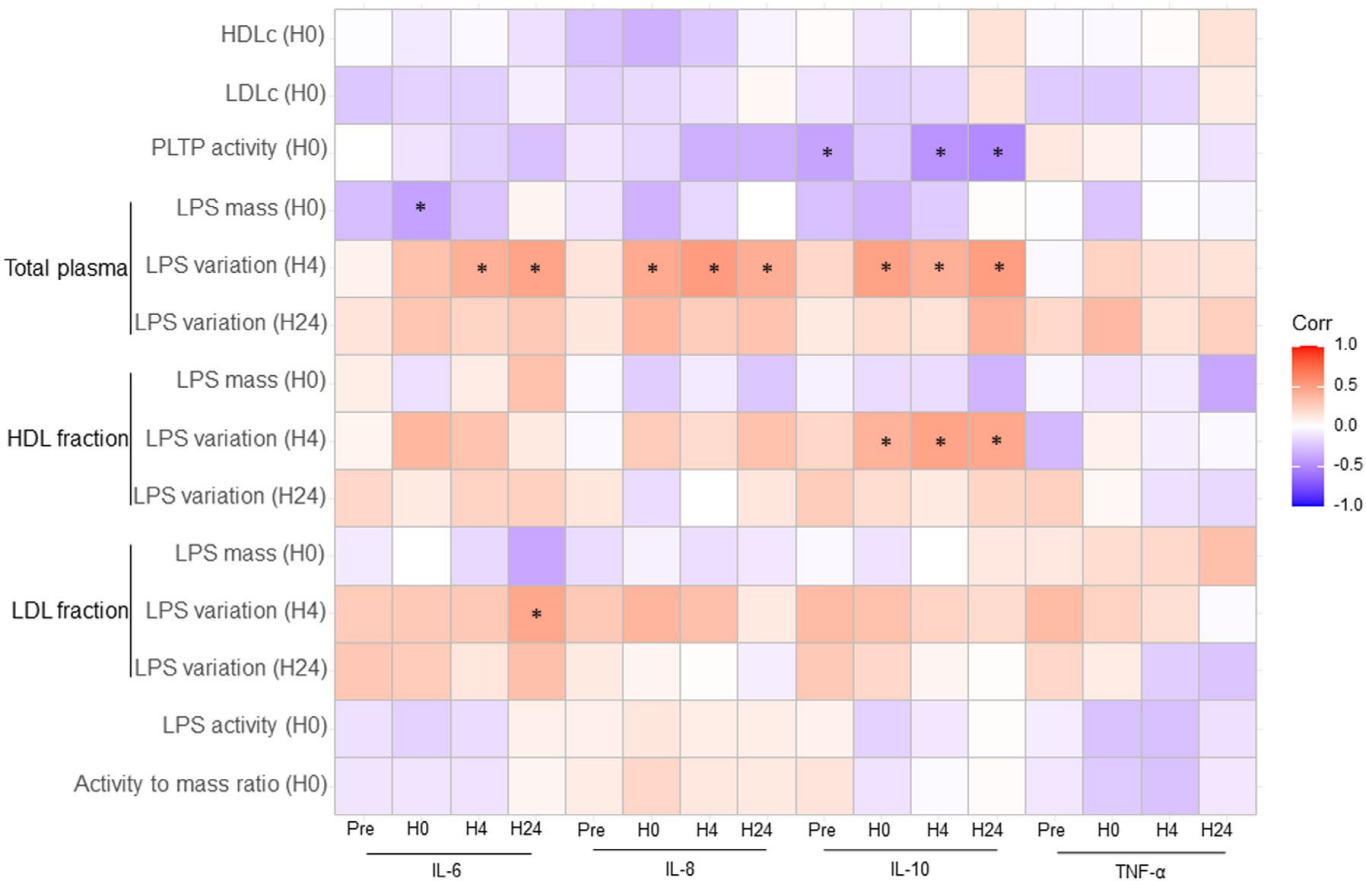
Correlations between lipoproteins, PLTP activity, the LPS burden and inflammation (cytokines: IL‐6, IL‐8, IL‐10 and TNF‐alpha). HDL, high density lipoproteins; IL, interleukin; LDL, low density lipoproteins; LPS, lipopolysaccharides; PLTP, phospholipid transfer protein; TNF, tumour necrosis factor. *Refers to significant correlations (*p* < 0.05, Spearman method, no correction for multiple testing).

Correlation of HDLc, PLTP activity and LPS with inflammatory biomarkers is presented in Figure 5. There was no significant correlation between HDLc and measured cytokines. PLTP activity was negatively correlated with IL‐10 at the pre‐operative time point, at H4 and H24. Increased H4 LPS elimination was correlated with reduced IL‐6, IL‐8 and IL‐10 levels. LPS mass and activity were not associated with increased inflammation.

## 4 DISCUSSION

Our results validate, in humans, previous findings obtained in vitro and in animal models on the role of lipoprotein in alleviating the LPS burden. Our data clearly illustrate the translation of this concept in patients with peritonitis and sepsis and put to the fore the tight connection between systemic and intraperitoneal HDL for LPS transport.

In gram‐negative infections, LPS causes a major inflammatory response which, if insufficiently regulated, leads to sepsis.^19^ In vitro and animal model experiments have demonstrated that the host could inactivate and eliminate LPS through their binding to lipoproteins. Nevertheless, although associations between HDL and mortality in sepsis^20^ and plasma LPS concentration and mortality in human peritonitis^21^ have been reported, the role of lipoprotein in alleviating the LPS burden has poorly been described in humans. In our data, a high proportion of LPS were bound to lipoproteins. High HDL cholesterol and PLTP activity were associated with accelerated LPS elimination. Accelerated LPS elimination was associated with reduced inflammation and mortality. Thus, by supporting the concept of lipoprotein‐driven LPS elimination in humans, our findings are an important step in the translational process. However, on the contrary to what we expected, LPS activity and mass were not associated with inflammation just after surgery, which may suggest that repetitive measurements are more informative than single measurements in this context of multifactorial inflammation. Because the prognosis of gram‐negative sepsis depends on the systemic inflammatory response, our findings might participate in the development of innovative lipoprotein‐based therapeutics. During sepsis, HDL metabolism is altered^22^ and HDL metabolism might be targeted for the treatment of endotoxemia, either by directly supplementing HDL or APO‐A1^3^, ^7^, ^23^, ^24^, ^25^ or by acting on PLTP with the aim of enhancing LPS transfer to lipoprotein.^10^, ^12^ Phospholipid supplementation has also been explored for endotoxin neutralization ability. Nevertheless, one large randomized controlled trial conducted in patients with gram‐negative sepsis failed to demonstrate the benefit of phospholipid supplementation upon survival.^26^

In our data, high HDLc was associated with reduced mortality but not with inflammation. PLTP activity was associated with reduced IL‐10 but not with mortality. Overall, this illustrates that the relationship between PLTP, HDL, enhanced LPS elimination, reduced inflammation and mortality is not that straightforward in humans. HDLs have pleiotropic effects, and even though in sepsis the main focus is on their immune‐regulating properties, part of their effect may be mediated by their numerous other properties (anti‐thrombotic, inhibition of adhesion molecule expression, anti‐apoptotic, antioxidants, for instance).^4^ Accordingly, some authors reported that during sepsis, the host response was poorly associated with the initial injury,^27^ suggesting that beyond initial pathogen recognition, activation of common secondary pathways might result in less specific patterns. In patients with low HDLc at ICU admission, LPS activity was higher at H4 and H24. Nevertheless, the activity to mass ratio was not different and LPS mass was reduced at H4 but not at H24. Because of the small sample size, we cannot infer whether the lower LPS activity was more related to higher elimination and/or inactivation in patients with high HDL.

Previous studies reported that LPS could be transferred between LDL and HDL^28^ and that LPS elimination could be driven by LDL receptors.^29^ The hypothesis of LPS transfer between HDL and LDL is indirectly supported hereby the fact that patients with high LPS concentrations in HDL had lower LPS concentrations in LDL. LPS transfer might also explain that patients with high HDLc had increased LPS elimination in the LDL fraction. We hypothesise that LPS bound to HDL and LDL might represent a common inactivated pool. From a broader perspective, these results suggest that LDL and HDL might be closely interconnected in the process of LPS binding and elimination.

Finally, our study explored for the first time lipoprotein parameters in the peritoneal fluid of patients with peritonitis. Our results showed a tight connection between systemic and intraperitoneal HDLc levels and HDL‐bound LPS, as well as with intraperitoneal PLTP activity. These observations are in line with our previous results in animal models of peritonitis showing an early binding of LPS to HDL at the site of infection.^11^ However, a higher HDLc level in peritoneal fluid was not associated with plasma LPS activity or mass, challenging the concept of LPS peritoneal clearance by HDL (although the analysis was power limited).

Some limitations need to be underlined. The small sample size limits the power of the analysis. In addition, we could not adjust for confounding conditions. However, our results are mostly concordant with previous experimental findings.^11^ Our data are observational; therefore, only associations can be inferred. The definition of peritonitis (with no clear cut‐off for white cell count in peritoneal fluid and no identification of a positive culture in each sample) is a limitation. We did not explore the qualitative modification of HDL, which might be a confounding condition between HDL quantity and function. We measured total 3OH fatty acids; thus, part of 3OH fatty acids measured might be human metabolites. However, LPS is the major part of 3OH fatty acids in humans; total 3OH fatty acids are highly associated with LPS levels (and probably to a greater extent in the context of severe peritonitis). Measuring Apolipoprotein A‐I could have added additional insight into HDL metabolism.

In conclusion, HDLc and PLTP activity were associated with accelerated (increased H4) endotoxin elimination in patients with peritonitis, thus supporting the translation of the reverse lipopolysaccharide transport to human gram‐negative sepsis. Higher H4 LPS elimination was associated with reduced inflammation and mortality. However, the link between HDL, PLTP, LPS elimination, reduced inflammation and improved outcome was not straightforward as LPS is not the only driver of inflammation in patients with sepsis. This is a small sample cohort study and further confirmation of our findings is needed.

## AUTHOR CONTRIBUTIONS

MN, BB, TG and DM contributed to the study design. MN, MA, VB, GP, SA, DL, JPPDB and PO collected the data. MN, BB, PGG and TG analysed the data. MN, PGG, BB and TG drafted the manuscript. All the authors have read, reviewed, edited the manuscript and approved the manuscript.

## FUNDING INFORMATION

This work was supported by the French National Research Agency under the program ‘Investissements d’Avenir’ with reference ANR‐11‐LABX‐0021 (LipSTIC Labex).

## CONFLICT OF INTEREST STATEMENT

MN: consulting honoraria and research grant from BAXTER, MN: formation fee Fresenius, MN: Congress fee Pfizer. PGG receives fees for lectures from Aguettant, AOP, BAXTER, Medtronic, Edwards and Vygon. PGG is a consultant for ABBOT. VB received fees for lectures from AOP and LFB.

## CONSENT TO PARTICIPATE

Informed consent was obtained from all patients or next of kin prior to inclusion.

## Supporting information


Appendix S1.

## Data Availability

Because indirect nominative data cannot be shared publicly under French laws, we cannot upload our minimal underlying data set. The datasets generated and/or analysed during the current study are not publicly available but are available from the corresponding author on reasonable request.
