# Supplementary material for: High‐density lipoproteins alleviate the endotoxin burden in patients with peritonitis and sepsis: The LIPS study
Source: Eur J Clin Invest. 2025 Jul 19;55(12):e70099. doi: 10.1111/eci.70099 (PMC12621300; doi:10.1111/eci.70099)
Supplement: Supplementary file 1 — Appendix S1. [file ECI-55-e70099-s001.docx]

**High density lipoproteins alleviate the endotoxin burden in patients with peritonitis and sepsis: The LIPS study**

Maxime Nguyen, Marvin Alvarez, Vivien Berthoud, Gaetan Pallot, Sohel Abagri, Damien Leleu, Jean-Paul Pais-De-Barros, Pablo Ortega Deballon, Pierre-Grégoire Guinot, David Masson, Thomas Gautier, Belaid Bouhemad

-

Supplemental data

|  | Pre-operative | H0 | H4 | H24 | Peritoneal |
| --- | --- | --- | --- | --- | --- |
| Triglycerides (mmol/l) | 1.56 [0.96;1.88] | 1.47 [1.08;1.89] | 1.55 [1.04;2.28] | 1.32 [1.05;2.69] | 1.02 [0.61;1.28] |
| HDLc (mmol/l) | 0.43 [0.31;0.91] | 0.51 [0.25;0.72] | 0.49 [0.22;0.68] | 0.38 [0.17;0.70] | 0.13 [0.13;0.35] |
| LDLc (mmol/l) | 0.71 [0.48;1.06] | 0.61 [0.38;0.99] | 0.57 [0.37;0.99] | 0.60 [0.44;0.78] | 0.36 [0.17;0.62] |
| PLTP activity (AU) | 37.7 (30.0) | 47.3 (30.1) | 51.6 (29.1) | 57.8 (28.3) | 0.00 [0.00;38.6] |

**Sup table 1**. Lipid parameters in the different biological samples

HDLc: High density lipoprotein cholesterol; LDLc: Low density lipoprotein cholesterol; PLTP: Phospholipid transfer protein.

Data are presented as median [Q1;Q3] or as mean (IQR).

|  | No bacterial identification  N = 13 | Bacterial identification  N = 14 | p-values |
| --- | --- | --- | --- |
| LPS mass (total 3OH , pmol/L) | | | |
| Pre-operative | 877 [618;1031] | 799 [625;919] | 0.497 |
| H0 | 742 [617;920] | 743 [623;793] | 0.734 |
| H4 | 808 [703;1025] | 672 [525;877] | 0.123 |
| H24 | 912 [723;1000] | 696 [625;809] | 0.115 |
| Peritoneal fluids | 10.0 [3.45;10.0] | 10.0 [2.31;10.0] | 1.000 |
| LPS activity (EU/ml) |  |  |  |
| Pre-operative | 0.34 [0.09;0.65] | 0.28 [0.15;1.00] | 0.496 |
| H0 | 0.42 [0.05;0.73] | 0.16 [0.06;0.58] | 0.575 |
| H4 | 0.35 [0.10;0.72] | 0.36 [0.06;0.83] | 0.836 |
| H24 | 0.13 [0.04;0.28] | 0.50 [0.04;1.44] | 0.180 |
| Peritoneal fluids | 10.0 [3.45;10.0] | 10.0 [2.31;10.0] | 1.000 |

**Sup table 2.** Lipopolysaccharide burden depending on bacterial identification

LPS: lipopolysaccharides.

Data are presented as median [Q1;Q3] or as mean (IQR). P values refer to between-group comparisons

|  | No-bacteriemia  N = 15 | Bacteriemia  N = 8 | p-values |
| --- | --- | --- | --- |
| LPS mass (total 3OH , pmol/L) | | | |
| Pre-operative | 740 [560;981] | 763 [664;878] | 0.846 |
| H0 | 682 [536;843] | 760 [693;803] | 0.478 |
| H4 | 758 [643;889] | 625 [487;828] | 0.219 |
| H24 | 802 [681;882] | 688 [616;703] | 0.176 |
| Peritoneal fluids | 775 [587;1969] | 1513 [1393;1739] | 0.439 |
| LPS activity (EU/ml) |  |  |  |
| Pre-operative | 0.16 [0.06;0.70] | 0.63 [0.22;1.21] | 0.174 |
| H0 | 0.20 [0.04;0.52] | 0.40 [0.15;1.07] | 0.285 |
| H4 | 0.23 [0.12;0.50] | 0.30 [0.04;0.76] | 0.731 |
| H24 | 0.36 [0.05;0.71] | 0.06 [0.04;1.04] | 0.577 |
| Peritoneal fluids | 4.29 [1.96;10.0] | 10.0 [10.0;10.0] | 0.090 |

**Sup table 3.** Lipopolysaccharide burden depending on bacteriemia

LPS: lipopolysaccharides.

Data are presented as median [Q1;Q3] or as mean (IQR). P values refer to between-group comparisons.


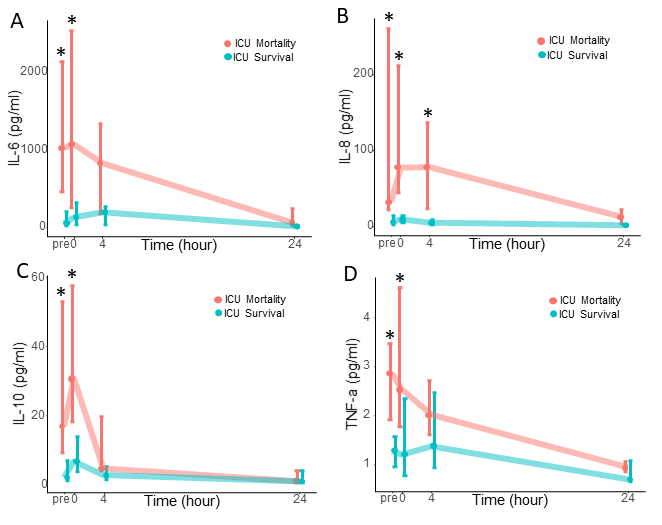


**Sup Fig 1.** Plasma cytokine concentration according to intensive care unit mortality

IL: Interleukin; TNF: Tumor necrosis factor; ICU: Intensive care unit

Data are presented as median and IQR [25;75]

*refers to significant between groups differences (p < 0.05, Wilcoxon tests, no correction for repeated testing)

**
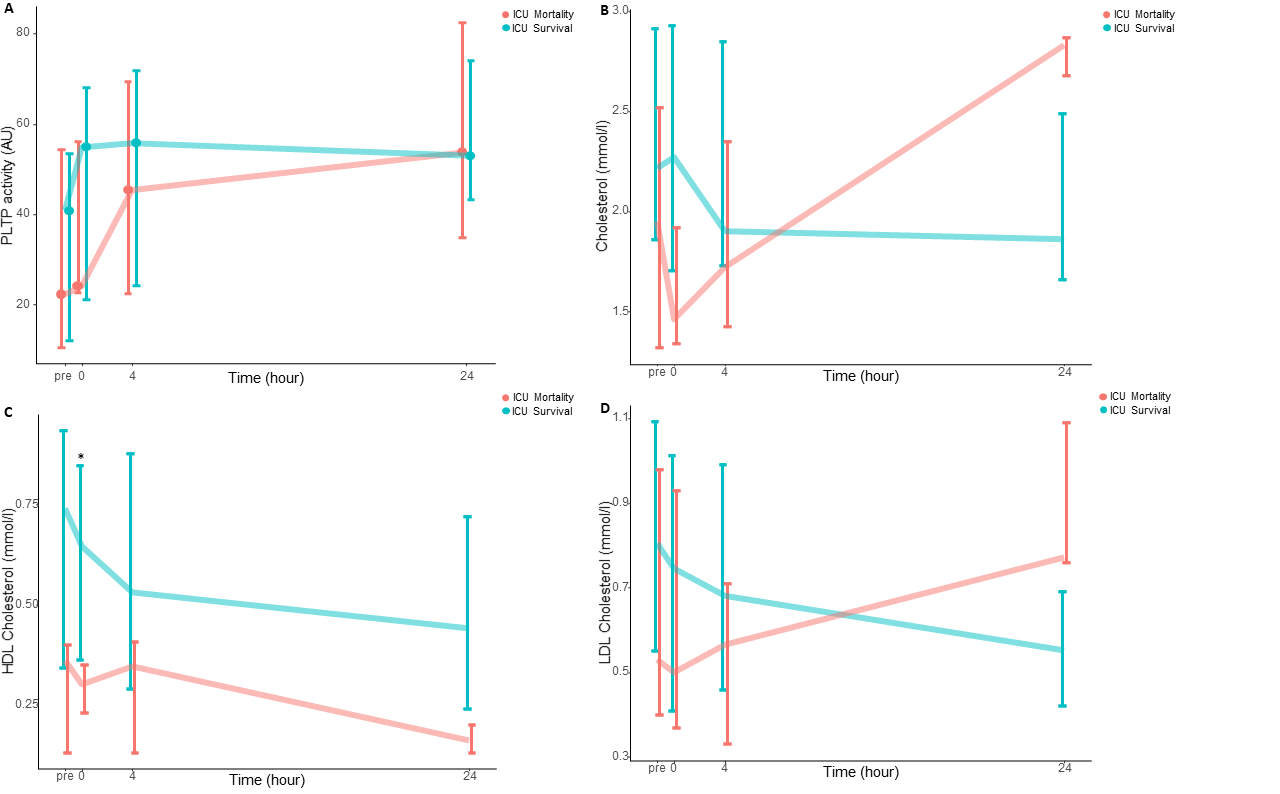
**

**Sup Fig 2.** kinetics for **(A)** PLTP activity**, (B)** cholesterol**, (C)** HDL cholesterol and **(D)** LDL cholesterol according to intensive care unit mortality

HDL: High density lipoprotein; LDL: Low density lipoprotein; PLTP: Phospholipid transfer protein; ICU: intensive care unit

Data are presented as median and IQR [25;75]

*refers to significant between groups differences (p < 0.05, Wilcoxon tests, no correction for repeated testing)


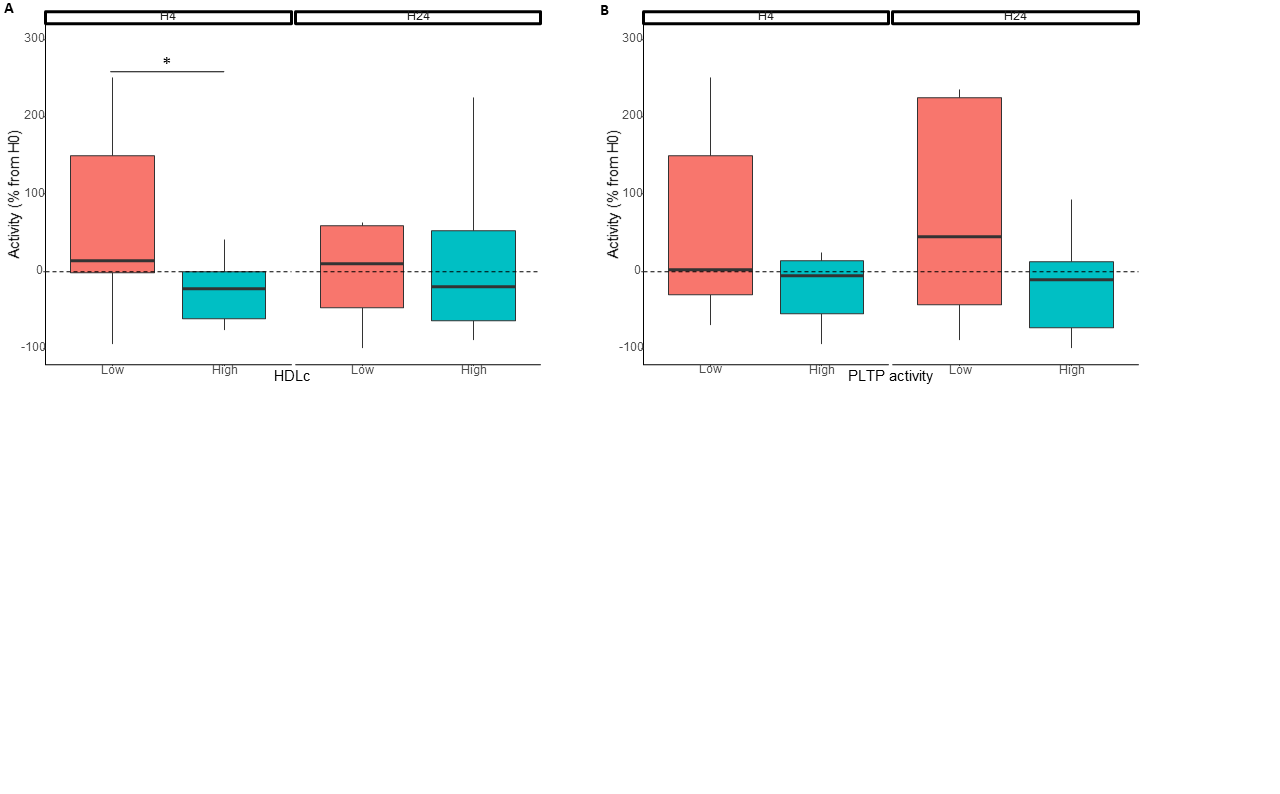


**Sup Fig 3.** Variations of LPS activity according to **(A)** HDLc plasma concentration **and (B)** PLTP activity.

HDLc: High density lipoprotein cholesterol; PLTP: Phospholipid transfer protein

*refers to significant between groups differences (p < 0.05, Wilcoxon tests, no correction for repeated testing)
